# Coexpression of human somatostatin receptor-2 (SSTR2) and SSTR3 modulates antiproliferative signaling and apoptosis

**DOI:** 10.1186/1750-2187-7-5

**Published:** 2012-05-31

**Authors:** Sajad A War, Ujendra Kumar

**Affiliations:** 1 Faculty of Pharmaceutical Sciences, The University of British Columbia, Vancouver, BC, V6T 1Z3, Canada

**Keywords:** Apoptosis, Photobleaching-Fluorescence resonance energy transfer (Pb-FRET), G protein-coupled receptor (GPCR), Heterodimerization, Somatostatin, Somatostatin receptors

## Abstract

**Background:**

Somatostatin (SST) via five G_i_ coupled receptors namely SSTR1-5 is known to inhibit cell proliferation by cytostatic and cytotoxic mechanisms. Heterodimerization plays a crucial role in modulating the signal transduction pathways of SSTR subtypes. In the present study, we investigated human SSTR2/SSTR3 heterodimerization, internalization, MAPK signaling, cell proliferation and apoptosis in HEK-293 cells in response to SST and specific agonists for SSTR2 and SSTR3.

**Results:**

Although in basal conditions, SSTR2 and SSTR3 colocalize at the plasma membrane and exhibit heterodimerization, the cell surface distribution of both receptors decreased upon agonist activation and was accompanied by a parallel increase in intracellular colocalization. Receptors activation by SST and specific agonists significantly decreased cAMP levels in cotransfected cells in comparison to control. Agonist-mediated modulation of pERK1/2 was time and concentration-dependent, and pronounced in serum-deprived conditions. pERK1/2 was inhibited in response to SST; conversely receptor-specific agonist treatment caused inhibition at lower concentration and activation at higher concentration. Strikingly, ERK1/2 phosphorylation was sustained upon prolonged treatment with SST but not with receptor-specific agonists. On the other hand, SST and receptor-specific agonists modulated p38 phosphorylation time-dependently. The receptor activation in cotransfected cells exhibits G_i_-dependent inhibition of cell proliferation attributed to increased PARP-1 expression and TUNEL staining, whereas induction of p21 and p27^Kip1^ suggests a cytostatic effect.

**Conclusion:**

Our study provides new insights in SSTR2/SSTR3 mediated signaling which might help in better understanding of the molecular interactions involving SSTRs in tumor biology.

## Background

G protein-coupled receptors (GPCRs) assemble as oligomers with distinct pharmacological, biochemical and physiological properties [1-3]. The concept of oligomeri-zation with efficacious changes in downstream signaling pathways have broadened the therapeutic potential of drugs targeting GPCRs. Somatostatin (SST) is a pleiotropic inhibitory peptide and regulates endocrine and exocrine secretions, neurotransmission and cell proliferation through five different receptor subtypes coupled to G_i_ proteins namely somatostatin receptors (SSTR1-5) [4,5]. SSTR subtypes display receptor-specific homo- and/or heterodimerization within the family and other GPCRs with unique signaling characteristics [3,6-9]. This notion is further supported by our previous studies demonstrating enhanced signaling properties in the heteromeric complex of human SSTR2 or SSTR5 with dopamine receptor-2 [10,11]. Interestingly, the heterodimer of SSTR2/SSTR3 of rat origin has been reported to abrogate SSTR3 functions [12]. However, rat SSTRs show distinct response to agonist in comparison to human SSTRs, [9,12-14].

All SSTR subtypes upon activation couple to G_i_ proteins and inhibit adenylyl cyclase (AC) in a pertussis toxin (PTX)-sensitive manner [4]. Importantly, in cells coexpressing human SSTR2 and SSTR5, cyclic adenosine monophosphate (cAMP) inhibition was enhanced upon activation of SSTR2 [3]. Conversely, cAMP levels remained unchanged in response to SSTR3-specific agonist in cells cotransfected with rat SSTR2 and SSTR3, whereas the activation of SSTR2 caused significant inhibition of cAMP [12]. We and others have shown the role of SSTRs in regulating intracellular signaling molecules including mitogen-activated protein kinases (MAPKs) which are implicated in cell proliferation, differentiation, migration and apoptosis [3,9,15-20]. The antiproliferative response of SSTRs is associated with the phosphorylation of selective downstream cascades including extracellular signal-regulated kinases (ERKs) depending upon the receptor subtype, cell environment and extracellular factors [3,4,8,9,21-23]. ERK is activated by SSTR1 and SSTR4, whereas inhibited upon activation of SSTR5 [8,24-26]. On the other hand, SSTR2 and SSTR3 exhibit dual effect on ERK phosphorylation in a cell-specific manner [4,9,12,22,25,27]. In the oligomeric complex of human SSTR2/SSTR5 or rat SSTR2/SSTR3, ERK1/2 phosphorylation has been attributed to SSTR2 activation [3,12]. Alterations in stress-related p38 MAPK have been frequently observed in human tumors and various other cell lines of tumor origin [28,29]. p38 signaling has diverse biological consequences including pro-/anti-apoptotic effects in a cell-dependent manner [27-29]. Importantly, the antiproliferative response mediated by SSTR2 but not SSTR3 has been associated with the activation of p38 MAPK [27]. Inhibition of cell proliferation by SSTR subtypes engages multiple converging mechanisms, however SSTR2 and SSTR3 are specifically linked to cell cycle arrest and apoptosis, respectively [3,4,9,30-35].

Agonist-induced internalization of SSTRs is time, temperature, and receptor-specific; however heterodimerization plays a determinant role on receptor trafficking [36,37]. Importantly, agonist-mediated internalization of SSTRs varies significantly between receptors of rat and human origin [9,12,13,36]. SSTR1 of rat origin internalized in response to agonist, whereas human SSTR1 rather up-regulated at the cell surface [36,38]. The constitutive homodimer of human SSTR2 dissociated into monomers at the plasma membrane prior to agonist-stimulated internalization, whereas rat SSTR2 internalized as homodimer [6,12]. On the other hand, human and rat SSTR3 internalized as homodimers upon agonist treatment [9,12]. Strikingly, rat SSTR3 internalized and subsequently recycled to the cell surface in response to agonist, whereas human SSTR3 was targeted to degradation [13,14,36]. More importantly, C-tail of human SSTR3 was not essential for receptor trafficking, conversely, mutations in C-terminal of rat SSTR3 abrogated the agonist-mediated internalization [9,13].

SSTR3 shares the least amino acid homology between rat and human species as compared to other SSTR subtypes [4]. Although, rat SSTR2/SSTR3 heterodimerization abolished the functions of SSTR3 [12], the same may not be speculated for human SSTR2 and SSTR3 as receptors of different origin have diverse signaling properties and need to be elucidated in detail. In this study, using morphological, biochemical and biophysical techniques, we provide first evidence for human SSTR2/SSTR3 heterodimerization in human embryonic kidney (HEK-293) cells and its implications in signaling and antiproliferation.

## Materials and methods

### Materials

Non-peptide agonists for SSTR2 (L-779976) and SSTR3 (L-796778) were kindly provided by Dr. S.P. Rohrer from Merck & Co [39]. SST was purchased from Bachem Inc., Torrance, CA, USA. HEK-293 cells were obtained from ATCC, Manassas, VA, USA. Rabbit polyclonal antibodies against SSTR2 were generated and characterized as described previously [40]. Mouse monoclonal antibodies against p21 and PARP-1 were purchased from BD-Biosciences, Mississauga, ON, Canada. Rabbit polyclonal antibodies for total and phospho-ERK1/2 and p38 were obtained from Cell Signaling Technology, Danvers, MA, USA. Fluorescein and rhodamine or peroxidase conjugated goat anti-mouse and goat anti-rabbit secondary antibodies were purchased from Jackson Immuno Research Laboratories, West Grove, PA, USA). Mouse monoclonal antibodies for p27^Kip1^ were obtained from Santa Cruz Biotechnology Inc., Santa Cruz, CA, USA. Mouse monoclonal antibodies against hemagglutinin (HA) and β-Tubulin were purchased from Sigma-Aldrich Inc., St. Louis, MO, USA. cAMP kit was obtained from BioVision Inc., Milpitas, CA, USA. TUNEL kit was purchased from La Roche Applied Science, Mannheim, Germany. ECL Western blotting detection kit and nitrocellulose Hy-Bond ECL membrane were purchased from GE Healthcare, Piscataway, NJ, USA. Protein A/G-Agarose beads were obtained from Calbiochem, EMD Biosciences, Darmstadt, Germany. Dulbecco’s modified eagle medium (DMEM), trypsin-EDTA, phosphate buffered saline (PBS) were purchased from Invitrogen, Burlington, ON, Canada. Reagents for electrophoresis were obtained from Bio-Rad Laboratories, Mississauga, ON, Canada. Other reagents were of AR grade and were purchased from various sources.

### Human SSTR2 and SSTR3 constructs and transfection

Human SSTR2 construct was prepared using pCDNA3.1/Hyg vector (hygromycin resistance). Constructs expressing human SSTR3 and N-terminal HA-tagged human SSTR3 (HA-SSTR3) were prepared using pCDNA3.1/Neo vector (neomycin resistance), as previously described [8,41]. HEK-293 cells which lack endogenous SSTRs were stably transfected with SSTR2 and SSTR3 using Lipofectamine transfection reagent as previously described [41]. Clones were selected and maintained in DMEM containing 10% fetal bovine serum (FBS) with 400 μg/ml hygromycin or 700 μg/ml neomycin or both in an incubator containing 5% CO_2_ at 37°C.

### Cell treatments

The concentrations for SST and receptor-specific agonists used in this study are as follows: SST (1, 50 nM and 1 μM); L-779976 (10 and 100 nM); L-796778 (25 and 50 nM). The binding affinity of SST for SSTR2 and SSTR3 is 0.2–1.3 nM and 0.3–1.6 nM, respectively. Furthermore, the binding affinity for L-779976 (SSTR2 agonist) and L-796778 (SSTR3 agonist) for their respective receptors is 0.05 nM and 24 nM, respectively [4]. Our previous studies demonstrated significant changes in dimerization and signaling molecules when SSTR subtypes were activated by different concentrations of SST and receptor-specific agonists [3,41,42]. Accordingly, in the present study we used the different concentrations of SST and receptor-specific agonists to understand the signaling aspects of SSTR2 and SSTR3.

### Co-immunoprecipitation (CO-IP) analysis

To determine the formation of heteromeric complex between SSTR2 and SSTR3, CO-IP was performed in mono- and/or cotransfectants expressing SSTR2 and SSTR3, and *wt-*HEK-293 cells. Briefly, 250 μg of total membrane protein prepared from untreated cells was solubilized in 1 ml of radioimmune precipitation assay (RIPA) buffer (150 mM NaCl, 50 mM Tris–HCl, 1% Nonidet P-40, 0.1% SDS, 0.5% sodium deoxycholate, protease and phosphatase inhibitors 1:100, pH 8.0) for 1 h at 4°C. Samples from cotransfected cells were then incubated overnight at 4°C with anti-HA antibody to immunoprecipitate SSTR3. To rule out detection of non-specific band, membrane fractions from monotransfectants expressing SSTR2 or SSTR3 were immunoprecipitated with anti-HA and anti-SSTR2 antibodies, respectively; whereas *wt-*HEK-293 were incubated with anti-SSTR2 antibody. On the following day, protein A/G-agarose beads (25 μl) were added to the samples for 2 h at 4°C to immunoprecipitate the antibody. The immunoprecipitate was fractionated on 7% SDS-PAGE and processed for Western blot analysis to detect SSTR2/SSTR3 heteromeric complex as previously described [43].

### Microscopic photobleaching-fluorescence resonance energy transfer (Pb-FRET) analysis for heterodimerization

To explore SSTR2 and SSTR3 heterodimerization, microscopic Pb-FRET analysis was performed in cotransfected cells as previously described [41]. Cells grown on poly-D-lysine coated glass coverslips were treated with SST (1, 50 nM and 1 μM), L-779976 (10 and 100 nM) and L-796778 (25 and 50 nM) or in combination for 10 min at 37°C. Post-treatment, cells were fixed in 4% paraformaldehyde (PF) for 15 min on ice. The coverslips were incubated at 4°C overnight with rabbit polyclonal anti-SSTR2 and mouse monoclonal anti-HA (for SSTR3) primary antibodies, followed by the addition of rhodamine-goat anti-rabbit (for SSTR2) and FITC-goat anti-mouse (for SSTR3) secondary antibodies to create donor-acceptor pair. Finally, the coverslips were processed for Pb-FRET analysis as previously described [8,9]. The FRET efficiency (E) was measured as a percent based upon photo bleaching time constants of the donor taken in the absence (D-A) and presence (D + A) of acceptor according to the formula E = [1-(D-A/D + A)] × 100.

### Immunocytochemistry for receptor colocalization and internalization

To analyze receptor colocalization and internalization, cotransfected cells were grown on poly-D-lysine coated glass coverslips and treated with different concentrations of SST and receptor-specific agonists as described in the previous section “Pb-FRET analysis” for 15 min at 37°C. Following fixation in 4% PF on ice, the cover slips were washed three times in PBS. Cells were permeabilized for intracellular expression in 0.3% Triton X-100 for 15 min at room temperature, whereas non-permeabilized cells were used for membrane expression. The incubation with primary and secondary antibodies was done as described in the previous section “Pb-FRET analysis”. After three washes in PBS, the coverslips were mounted on glass slides and analyzed under Leica fluorescence microscope as previously described [44]. Adobe Photoshop and Image J software, NIH were used for making the composites.

### cAMP assay

Mono- and/or cotransfected cells expressing SSTR2 and SSTR3 were seeded in 6-well plates and grown to 70% confluency, and cAMP assay was performed as previously described [8]. Briefly, cells were pre-treated for 30 min with 3-isobutyl-1-methylxanthine (0.5 mM) to prevent cAMP degradation. Cells were then treated for 30 min with SST and receptor-specific agonists in the presence of 20 μM forskolin (FSK). DMEM was used as control. Cells were scraped in 0.1 N HCl, normalized for protein amount and cAMP was determined by immunoassay using a cAMP kit following the manufacturer’s instructions.

### Western blot analyses for signaling

For each experiment, 10000 cells were seeded per culture flask and grown to 80% confluency. For ERK1/2 and p38, cells were treated with SST and receptor-specific agonists for 30 min and 24 h, respectively with or without 24 h serum starvation (no FBS in culture media). To determine the expression of PARP-1, p21 and p27^Kip1^, cells were treated with SST and receptor-specific agonists for 24 h with or without serum deprivation. Post-treatment, cells were lysed in RIPA buffer and the cell lysate was fractionated on SDS-PAGE and probed for total and phospho ERK1/2 and p38 (1:1000), PARP-1 (1:5000), p21 and p27^Kip1^ (1:750) using specific antibodies as previously described. β-Tubulin (1:10000) was used as loading control. All other procedures like membrane blocking, primary and secondary antibody incubation, and chemiluminescence detection were performed as previously described [9]. Immunoblots were visualized with an Alpha Innotech FluorChem 8800 gel box imager (Protein Simple, Santa Clara, CA, USA) and densitometric analysis was performed by using FluorChem software (Protein Simple, Santa Clara, CA, USA).

### Cell proliferation assay

Cell proliferation was determined by MTT assay as previously described [3,9]. Mono- and/or cotransfected cells expressing SSTR2 and SSTR3 were serum starved for 24 h. Cells were either subjected to pre-incubation with PTX (100 ng/ml) for 18 h, or directly treated with different concentrations of SST and receptor-specific agonists for 24 h in DMEM containing FBS before processing for MTT assay. Briefly, 20 μl of 5 mg/ml MTT solution in DMEM was added and incubated for 2 h at 37°C. The formazan crystals formed were dissolved in 200 μl of isopropanol and the absorbance was measured in a microplate spectrophotometer at 550 nm.

### TUNEL staining to detect apoptosis

Cells coexpressing human SSTR2 and SSTR3 were grown on poly-D-lysine coated glass coverslips and treated with different concentrations of SST and receptor-specific agonists for 24 h. Cells were fixed in 4% PF and rinsed three times with PBS. After permeabilization in solution containing 0.1% Triton X-100 and 0.1% sodium citrate, cells were washed in PBS followed by incubation with TUNEL reaction mixture for 1 h at 37°C in dark. Finally, the coverslips were washed in PBS and mounted on glass slides for analysis under Leica fluorescence microscope. Adobe Photoshop was utilized for making composites.

### Statistical analysis

Results are expressed as mean ± S.D of three independent experiments. Statistical analysis was done by one- or two-way ANOVA and *post hoc* Dunnett’s or Bonferroni’s tests, as applicable. GraphPad Prism 4.0 (GraphPad Software, Inc., La Jolla, CA, USA) was used for performing data analysis and *p* value < 0.05 was considered statistically significant.

## Results

### Human SSTR2 and SSTR3 exist as constitutive heterodimer at cell surface

**Figure 1:**
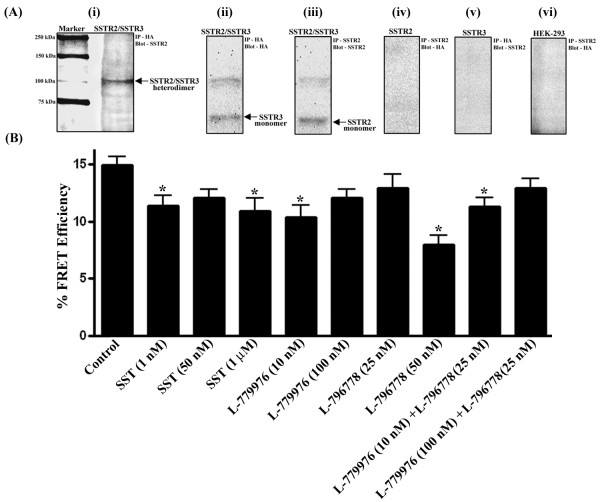
**SSTR2/SSTR3 exhibit heterodimerization at cell surface A (i).** Membrane extracts prepared from cotransfected cells were immunoprecipitated with HA antibody (SSTR3) and probed for SSTR2 as described in Materials and methods. The receptor-specific band for the heteromeric complex of SSTR2/SSTR3 was detected at the expected size of ~117 kDa. **A (ii-iii).** The membranes were reprobed to detect SSTR3 and SSTR2 monomers, respectively. **A (iv-vi)**. The specificity of the heterodimer was confirmed in HEK-293 cells and monotransfectants expressing SSTR2 and SSTR3 **B.** Cotransfected cells were treated with SST (1, 50 nM and 1 μM), L-779976 (10 and 100 nM) and L-796778 (25 and 50 nM) or in combination for 10 min at 37°C and subjected to microscopic Pb-FRET analysis. Histogram represents relative FRET efficiency of 14.9% in control suggesting the presence of SSTR2/SSTR3 heterodimers. However, the heterodimer is stable upon treatment with SST and receptor-specific agonists, albeit with decreased relative FRET efficiency. Results are expressed as mean ± S.D of three independent experiments.

To investigate whether human SSTR2 and SSTR3 exist in a heteromeric complex, CO-IP was performed in cotransfected cells expressing SSTR2 (279 ± 28 fmol/mg protein) and SSTR3 (285 ± 31 fmol/mg protein). As illustrated in Figure 1A (i), SSTR2 is expressed in the SSTR3 immunoprecipitate at the expected molecular size of ~117 kDa. The specificity of the oligomeric complex was confirmed by the absence of heterodimer band in monotransfectants and *wt*-HEK-293 cells under the same experimental conditions [Figure 1A (iv-vi)]. Upon probing SSTR3 immunoprecipitate with anti-HA antibody, a band corresponding to SSTR3 monomers was observed at ~60 kDa [Figure 1A (ii)]. In addition, SSTR2 monomers were detected at ~57 kDa after SSTR2 immunoprecipitate was probed with anti-SSTR2 antibody [Figure 1A (iii)]. To further validate the data from CO-IP, microscopic Pb-FRET analysis was performed in cotransfected cells following treatment with different concentrations of SST and receptor-specific agonists. As depicted in Table 1 and Figure 1B, SSTR2 and SSTR3 assembled as heterodimers at the cell surface in basal conditions and displayed a high relative FRET efficiency of 14.9 ± 1.2%. Moreover, upon treatment with SST (1 nM and 1 μM), SSTR2-specific agonist (10 nM), and SSTR3-specific agonist (50 nM) or in combination, the effective FRET efficiency was significantly decreased when compared to control (*, p < 0.05). Importantly, the combination of receptor-specific agonists displayed comparable FRET efficiencies with SST which activates SSTR2 and SSTR3 equally. Taken together, these results confirm that SSTR2/SSTR3 exist in a heteromeric complex, however the relative FRET efficiency was sufficient to support heterodimerization even upon receptors activation.

**Table 1:** Photobleaching time-constants and relative-FRET efficiencies in HEK-293 cells coexpressing SSTR2 and SSTR3

*Treatment*	*τ*_*avg*_*(s)*		*n*	*E (%)*
Control	D - A	18.3 ± 0.5	55	14.9 ± 1.2
	D + A	21.5 ± 0.7	54	
SST (1 nM)	D - A	18.6 ± 0.4	58	11.4 ± 1.4*
	D + A	21 ± 0.5	56	
SST (50 nM)	D - A	18.8 ± 0.4	59	12.1 ± 1.1
	D + A	21.4 ± 0.4	60	
SST (1 μM)	D - A	19.6 ± 0.6	57	10.9 ± 1.6*
	D + A	22 ± 0.2	55	
L-779976 (10 nM)	D - A	20.7 ± 0.3	56	10.4 ± 1.5*
	D + A	23.1 ± 0.3	59	
L-779976 (100 nM)	D - A	20.3 ± 0.4	58	12.1 ± 1.2
	D + A	23.1 ± 0.4	54	
L-796778 (25 nM)	D – A	19.5 ± 0.9	59	12.9 ± 1.7
	D + A	22.4 ± 0.7	54	
L-796778 (50 nM)	D - A	20.8 ± 0.5	60	8 ± 1.2*
	D + A	22.6 ± 0.6	57	
L-779976 (10 nM) + L-796778 (25 nM)	D - A	19.6 ± 0.5	55	11.3 ± 0.9*
	D + A	22.1 ± 0.2	54	
L-779976 (100 nM) + L-796778 (25 nM)	D - A	19.6 ± 0.5	57	12.9 ± 1.4
	D + A	22.5 ± 0.5	54	

D − A and D + A correspond to donor without and with acceptor, respectively; *τ*_*avg*_ refers to average of *n* photobleaching time constants; *n* is the total number of cells analyzed; *E (%)* is mean relative FRET efficiency. Mean ± SD is representative of three independent experiments (*, p < 0.05).

### SSTR2 and SSTR3 colocalize at the cell surface

**Figure 2:**
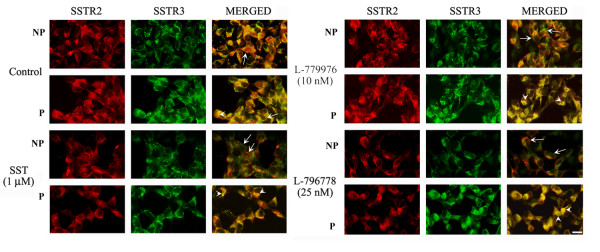
**Representative photomicrographs illustrating fluorescence analysis and internalization of SSTR2 and SSTR3 upon agonist treatment.** Cotransfected cells were treated with SST (1 μM), L-779976 (10 nM) and L-796778 (25 nM) for 15 min at 37°C, and processed for immunocytochemistry as described in Materials and methods. Fluorescent microscopic images show distribution of SSTR2 (red), SSTR3 (green) and colocalization (yellow) at the cell surface and intracellularly. SSTR2 and SSTR3 display colocalization at the cell surface and internalize upon treatment with SST and specific agonists. Note that activation of one receptor caused a significant down-regulation of both receptors at cell surface. Arrows and arrowheads in the merged panel represent membrane and cytosolic expression, respectively. Scale bar = 10 μm. Data are representative of mean ± S.D of three independent experiments.

As previously described, rat SSTR2/SSTR3 complex dissociated upon treatment with SST leading to SSTR2 internalization whereas the endocytosis of SSTR3 was impaired [12]. To ascertain whether the same holds true for human SSTR2 and SSTR3, we performed immunocytochemistry in cotransfected cells. As shown in Figure 2, SSTR2 and SSTR3-like immunoreactivity and colocalization at the cell surface was decreased in response to SST, accompanied by a parallel increase in cytosolic expression. Most importantly, the independent activation of SSTR2 or SSTR3 resulted in the loss of immunoreactivity for both receptors at the plasma membrane, suggesting a potential role for heterodimerization in regulating receptor trafficking.

### Agonist mediated cAMP inhibition by SSTR2/SSTR3

**Figure 3:**
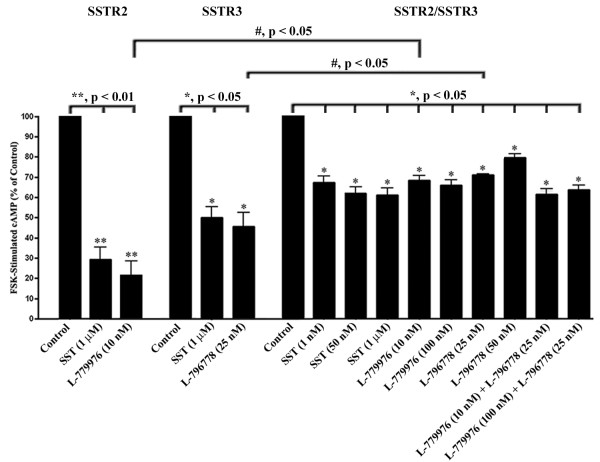
**cAMP inhibition upon activation of SSTR2/SSTR3.** Mono- and/or cotransfected expressing SSTR2 and SSTR3 were pre-treated with 3-isobutyl-1-methylxanthine (0.5 mM) for 30 min followed by addition of FSK (20 μM) in the presence or absence of SST (1, 50 nM and 1 μM), and receptor-specific agonists L-779976 (10 and 100 nM) and L-796778 (25 and 50 nM), or as indicated for 30 min at 37°C and processed for cAMP assay. Note the significant inhibition of FSK-stimulated cAMP in the presence of SST and receptor-specific agonists; an indication that SSTR2/SSTR3 complex in cotransfected cells is functionally active, albeit at a lesser degree than the monotransfectants. Data represent mean ± S.D of three independent experiments.

The decreased cAMP accumulation via negative regulation of AC upon SSTRs activation gives an unswerving estimate of receptor functionality [4]. We next examined cAMP levels in mono- and/or cotransfected cells expressing SSTR2 and SSTR3. As illustrated in Figure 3, SST and the receptor-specific agonists significantly inhibited cAMP in cotransfected cells when compared to control (*, p < 0.05). As expected, cAMP levels upon treatment with SST were comparable with the combination of receptor-specific agonists. In monotransfected cells, SST and receptor-specific agonists significantly inhibited cAMP (**, p <0.01; *, p < 0.05). Although, the levels of cAMP inhibition upon treatment with receptor-specific agonists were significantly decreased in cotransfected *vs.* monotransfected cells (#, p < 0.05), the receptor complex still demonstrated significant G_i_ coupling to AC.

### Time and receptor-dependent regulation of MAPK signaling by SSTR2/SSTR3

**Figure 4:**
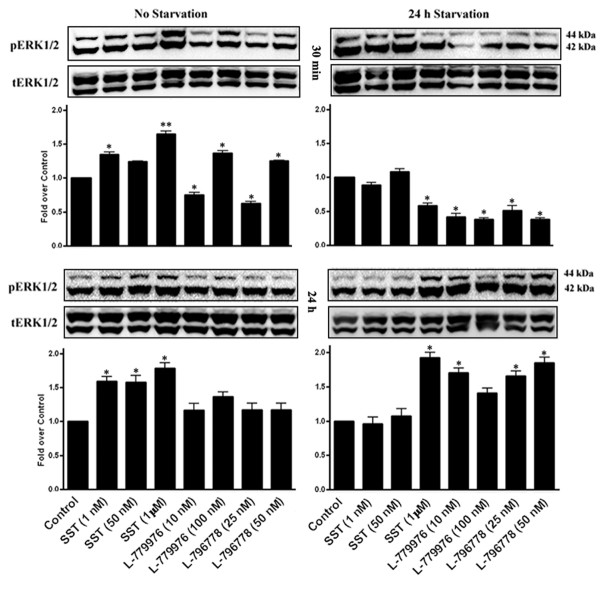
**Concentration and time-dependent modulation of ERK1/2 phosphorylation by SSTR2/SSTR3.** Cells stably cotransfected with SSTR2 and SSTR3 were treated with SST (1, 50 nM and 1 μM), L-779976 (10 and 100 nM) and L-796778 (25 and 50 nM) for 30 min (*top panel*) or 24 h (*bottom panel*) with or without FBS, and processed for total and pERK1/2 using Western blot analysis. Note a distinct pattern of ERK1/2 phosphorylation in response to SST and receptor-specific agonists in a concentration and time-dependent manner. Histograms represent the densitometric analysis and the results are presented as the ratio of phospho- and total-ERK1/2 expressed as fold over control. Mean ± S.D is representative of three independent experiments.

Our recent studies suggested agonist and time-dependent modulation of pERK1/2 in cells expressing SSTR2, SSTR3 or SSTR2/SSTR5 [3,9]. Using time-course experiments (30 min and 24 h), we examined the effect of heterodimerization on ERK1/2 phosphorylation in cotransfected cells with or without FBS. As depicted in Figure 4, the levels of pERK1/2 were significantly increased at lower and higher concentrations of SST for 30 min; whereas in response to receptor-specific agonists, ERK1/2 phosphorylation decreased at lower concentration and increased at higher concentration when compared to control (*, p < 0.05; **, p < 0.01). In the absence of FBS, pERK1/2 was significantly decreased by SST and receptor-specific agonists in comparison to control (*, p < 0.05). SST treatment for 24 h increased pERK1/2 when compared to non-treated cells (*, p < 0.05), whereas no significant changes were observed with receptor-specific agonists alone suggesting that co-activation of both receptors is a prerequisite for sustained ERK1/2 signaling. However in FBS-deficient conditions, SST and receptor-specific agonists significantly enhanced pERK1/2 in comparison to control (*, p < 0.05).

**Figure 5:**
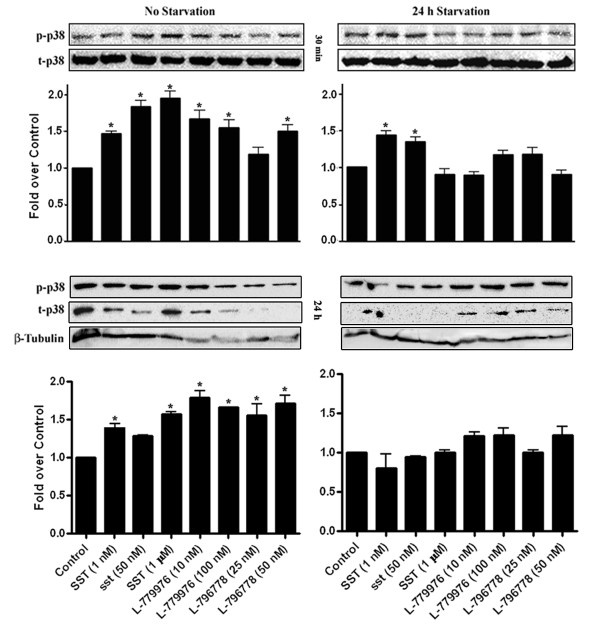
**Modulation of p38 phosphorylation in response to SSTR2/SSTR3 activation.** Cotransfected cells were treated with SST (1, 50 nM and 1 μM), L-779976 (10 and 100 nM) and L-796778 (25 and 50 nM) for 30 min (*top panel*) or 24 h (*bottom panel*) in the presence or absence of FBS, and processed for total and p-p38 using Western blot analysis. p38 phosphorylation increased upon treatment with SST and receptor-specific agonists for 30 min. In serum-deprived conditions, SST significantly increased p38 activation, whereas the effects of independent receptor activation were comparable to control. Prolonged treatment with SST and receptor-specific agonists significantly increased p-p38 levels, whereas in serum-deficient conditions, no significant changes were observed between treatment and control. Histograms represent the densitometric analysis and the results are presented as the ratio of phospho- and total-p38 (*top panel*) or phospho-p38 and β-Tubulin (*bottom panel*) expressed as fold over control. Mean ± S.D is representative of three independent experiments.

As illustrated in Figure 5, p38 phosphorylation was enhanced in cotransfected cells treated with SST and receptor-specific agonists for 30 min when compared to control (*, p < 0.05). SST significantly increased p38 phosphorylation in serum-deprived cells, whereas the inde-pendent receptor activation had no effect in comparison to control (*, p < 0.05). Upon prolonged treatment with SST and receptor-selective agonists, p38 signaling was enhanced when compared to control (*, p < 0.05). However no discernable changes in p-p38 levels were observed between treated and untreated cells under serum deprived conditions. Collectively, our results demonstrate distinct pattern of MAPK modulation upon agonist treatment in cotransfected cells.

### Inhibition of cell proliferation by SSTR2/SSTR3

**Figure 6:**
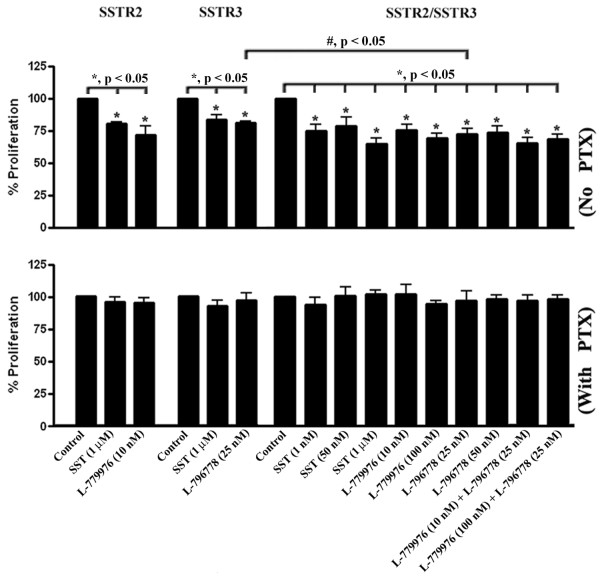
**The antiproliferative activity of SSTR2/SSTR3.** Mono- and cotransfected cells expressing SSTR2 and SSTR3 in the absence (*top panel*) and presence (*bottom panel*) of PTX were subjected to treatment with SST (1, 50 nM and 1 μM), L-779976 (10 and 100 nM) and L-796778 (25 and 50 nM) or as indicated for 24 h and processed for MTT assay. SST and receptor-specific agonists significantly inhibited cell proliferation in cotransfected cells in a PTX-sensitive manner. Note an increased antiproliferative effect of SSTR3-specific agonist in cotransfected cells when compared to monotransfectants expressing SSTR3. Data are presented as mean ± S.D of three independent experiments.

To determine the antiproliferative effects upon activation of SSTR2/SSTR3 by SST and receptor-specific agonists, MTT assay was accomplished in monotransfectants expressing SSTR2 or SSTR3 and cotransfected cells. Importantly, cells were treated in the presence or absence of PTX pre-treatment to elucidate a functional relationship between G_i_ coupling and antiproliferation. As depicted in Figure 6 (*top panel*), SST and the receptor-specific agonists resulted in significant inhibition of cell proliferation in cotransfected cells (*, p < 0.05). Furthermore, the combination of receptor-specific agonists exhibited a similar antiproliferative response to SST. In addition, monotransfectants expressing SSTR2 displayed a comparable inhibition of cell proliferation with cotransfected cells upon treatment with SSTR2-specific agonist. Strikingly, the effect of SSTR3-specific agonist was significantly higher in cotransfected cells in comparison to monotransfectants (28.6 ± 4.3% *vs.* 18.9 ± 1.6%; #, p < 0.05), suggesting the enhanced antiproliferative functions of SSTR3 in the heteromeric complex. Moreover, SSTR2 and SSTR3 mediated antiproliferative effects in mono- and cotransfected cells were G_i_-dependent as evident by no significant changes in cell proliferation upon receptor activation in cells pre-treated with PTX (Figure 6, *bottom panel*). Taken together, our data from MTT assay suggest a role played at least in part by receptor heterodimerization in mediating the antiproliferative effects of SST and receptor-specific agonists in cotransfected cells.

### Pro-apoptotic role of SSTR2 and SSTR3 in cotransfected cells

**Figure 7:**
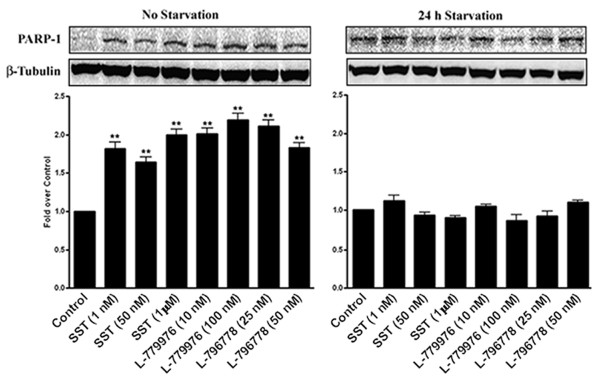
**The cytotoxic role for SSTR2/SSTR3.** Stable cotransfectants of SSTR2 and SSTR3 were subjected to 24 h treatment with SST (1, 50 nM and 1 μM), L-779976 (10 and 100 nM) and L-796778 (25 and 50 nM) in the presence or absence of serum-starvation and processed for PARP-1 expression by Western blot analysis. Cells treated with SST and receptor-specific agonists displayed increased expression of PARP-1. Note a high basal PARP-1 expression in serum-deprived conditions with no significant changes in response to SST and receptor specific agonists. β-Tubulin was used as a loading control. Data represent mean ± S.D of three independent experiments.

To further identify the putative mechanisms for the antiproliferative signal in cotransfectants, we determined the expression of PARP-1 for apoptosis. As illustrated in Figure 7, SST and the receptor-specific agonists significantly increased the expression of PARP-1 in comparison to control suggesting a cytotoxic effect (**, p < 0.01). However, high basal PARP-1 levels were observed in cells deprived of FBS which were maintained upon treatment with SST and receptor-specific agonists. These observations correlate with p38 signaling and might suggest a role for p38 MAPK in SSTR2/SSTR3 mediated apoptosis.

**Figure 8:**
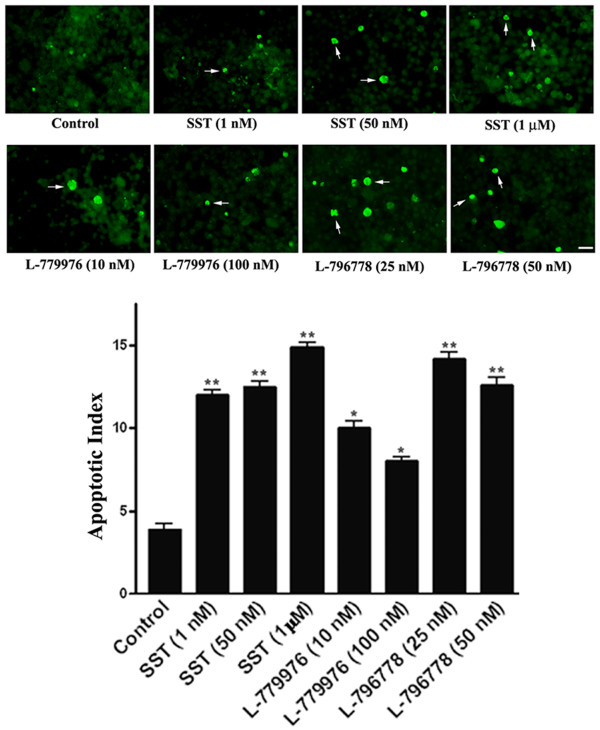
**Representative photomicrographs illustrating TUNEL positive cells as an index of apoptosis.** HEK-293 cells cotransfected with SSTR2/SSTR3 were treated with SST (1, 50 nM and 1 μM), L-779976 (10 and 100 nM) and L-796778 (25 and 50 nM) for 24 h, and processed for TUNEL staining. SST and receptor-specific agonists increased TUNEL labeling indicating cytotoxic role for SSTR2 and SSTR3. Histogram represents quantitative analysis of apoptotic cells. A total of 600–750 cells were counted for each treatment. Arrows in representative panels indicate apoptotic cells. Scale bar = 10 μm. Mean ± S.D is representative of three independent experiments.

To support the enhanced PARP-1 expression, *in situ* TUNEL assay was performed to quantify apoptosis in cotransfected cells expressing SSTR2/SSTR3. As depicted in Figure 8, SST and the receptor-specific agonists significantly increased the number of TUNEL-positive cells in comparison to control (*, p < 0.05; **, p < 0.01). Taken together, these findings show the cytotoxic role for SSTR2/SSTR3 upon activation.

### Induction of cell cycle arrest upon activation of SSTR2 and SSTR3

**Figure 9:**
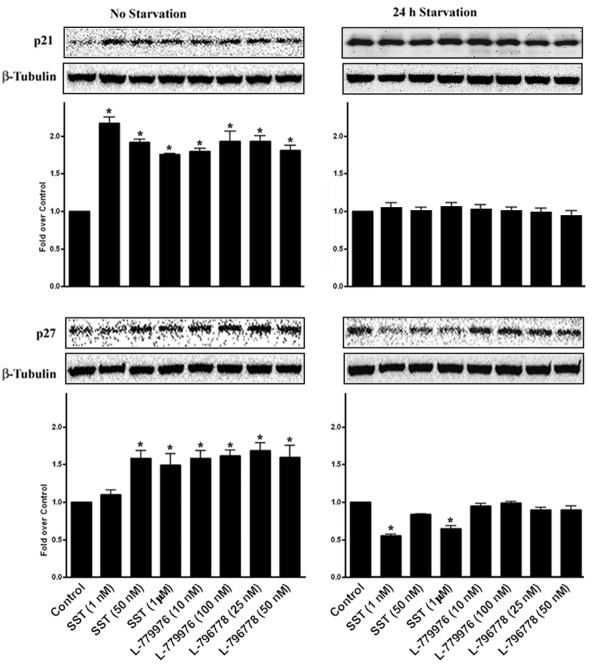
**p21 and p27**^**Kip1**^**mediated cytostatic effects upon activation of SSTR2/SSTR3.** Cotransfected cells were treated with SST (1, 50 nM and 1 μM), L-779976 (10 and 100 nM) and L-796778 (25 and 50 nM) for 24 h with or without serum starvation and processed for p21 (*top panel*) and p27^Kip1^ (*bottom panel*) expression using Western blot analysis. Induction of p21 and p27^Kip1^ in response to SST and receptor-selective agonists suggests a cytostatic role for both receptors. The high basal levels of p21 observed in serum-deprived cells were maintained upon treatment with SST and specific agonists. Conversely, SST decreased p27^Kip1^ expression in cotransfected cells by ~50% in FBS-deficient conditions. β-Tubulin was used as a loading control. Results are expressed as mean ± S.D of three independent experiments.

Earlier studies have revealed that SSTR2 but not SSTR3 mediated induction of cyclin-dependent kinase inhibitors p21 and p27^Kip1^ leads to cell cycle arrest [3,27,30,45]. To confirm whether SSTR2/SSTR3 complex alters the antiproliferative nature of native receptors, we next determined p21 and p27^Kip1^ expression to attest the role of receptors for cytostatic activity. As illustrated in Figure 9 (*top panel*), SST and receptor-specific agonists significantly increased p21 expression upto ~2 fold in comparison to control, suggesting a cytostatic effect exerted by SSTR2 and SSTR3 (*, p < 0.05). In the absence of FBS, the high basal expression of p21 was maintained upon receptors activation. Furthermore, p27^Kip1^ expression increased upto ~1.5 fold upon treatment with SST and receptor-specific agonists when compared to control. Conversely, in serum-deprived conditions, SST decreased the expression of p27^Kip1^ upto ~50%, whereas the effects of independent receptor activation were comparable to control (*, p < 0.05).

## Discussion

Several lines of evidence have described the role of GPCR oligomerization in the modulation of intracellular signaling cascades. However, the underlying molecular mechanisms are complex and unclear, and have only been partly elucidated to date. In the present study, we provide detailed description of functional analysis of human SSTR2 and SSTR3 heterodimerization in HEK-293 cells including receptor surface expression, internalization, MAPK signaling, cell proliferation and apoptosis in response to SST and receptor-specific agonists. To the best of our knowledge, this is the first comprehensive demonstration of a functional crosstalk between human SSTR2 and SSTR3.

We previously reported that human SSTR2 and SSTR3 exist as preformed homodimers in monotransfected cells and respond to agonist treatment in receptor-specific manner [6,9]. Our earlier studies also showed that homodimers of SSTR5 and heterodimers of SSTR4/SSTR5 are stable upon agonist activation [8,42]. We and others also demonstrated that SST induced dissociation of human SSTR2 homodimer and rat SSTR2/SSTR3 heterodimer at the cell surface [6,12]. Using CO-IP and microscopic Pb-FRET analysis, we here report that human SSTR2 and SSTR3 exist in a constitutive heteromeric complex at the plasma membrane. The receptor complex tends to remain stable upon treatment with SST and receptor-specific agonists with FRET efficiency sufficient to support heterodimerization. Nonetheless, the decrease in relative FRET efficiency might be attributed to the changes in receptor conformation and orientation at the cell surface upon agonist activation which cannot be excluded from the discussion.

We previously showed that agonist-induced dissociation of human SSTR2 homodimers into monomers at plasma membrane is a prerequisite for receptor internalization, whereas human SSTR3 internalized as homodimer [6,9]. Our immunocytochemistry data illustrate decreased cell surface distribution of SSTR2 and SSTR3 upon receptors activation. Strikingly, the independent activation of SSTR2 or SSTR3 resulted in a dramatic down-regulation of both receptors and decreased colocalization at plasma membrane. It is likely that the receptors internalize as heterodimers, which in part might be a plausible explanation for an increased colocalization in cytosol. These data contradict an earlier report where rat SSTR2/SSTR3 heterodimer, while promoting SSTR2 internalization, was shown to abrogate agonist-mediated endocytosis of SSTR3 [12]. However, a follow-up study from the same group revealed that the activation of SSTR2 in the heteromeric complex of SSTR2/μ-opioid receptor of rat origin promoted co-internalization of both receptors [37]. Accordingly, our results for human SSTR2/SSTR3 reinforce the concept that heterodimerization confers unique species-selective properties to native receptors.

SSTR subtypes elicit their cellular actions by inhibiting second messenger cAMP through PTX-sensitive G_i_ proteins, and oligomerization plays a key role in modulating such effects [4,10-12,41]. Our data demonstrate significant inhibition of cAMP in response to SST and receptor-specific agonists; however the G_i_ coupling was attenuated in cotransfectants when compared to monotransfected cells expressing SSTR2 or SSTR3. It is not clear whether the blunted G_i_ coupling in cotransfected cells is linked to the decreased FRET efficiencies in response to receptors activation. Moreover, it remains elusive whether the determinant role on cAMP inhibition in cotransfected cells is mediated by monomeric, homodimeric or heteromeric sub-populations. Whether the loss of G_i_ coupling in cotransfectants is linked to downstream signaling pathways is not well understood and future studies are warranted to delineate the molecular mechanisms involved.

Several previous studies have shown a significant role for MAPKs in cell survival, proliferation and apoptosis [15-18]. The cytostatic role for SSTR2 has been intimately associated with the modulation of ERK1/2 signaling in a cell-dependent manner [22,46,47]. Our previous study showed a robust increase in ERK1/2 phosphorylation upon transient activation of SSTR2 in monotransfected cells [3]. Interestingly, ERK1/2 remained in phosphorylated form upon prolonged activation of SSTR2 in cells coexpressing SSTR2/SSTR5, an effect attributed to receptor heterodimerization. We and others have previously shown that the activation of SSTR3 leads to cell cycle arrest or apoptosis depending upon the cell-type, however the cytotoxic role of SSTR3 was associated with pERK1/2 inhibition upon short-term agonist exposure [9,35]. Serum and growth factors have also been shown to stimulate ERK activation and cell proliferation in astrocytes and CHO-K1 cells expressing SSTR1 [48,49]. In the present study, although ERK1/2 modulation by SST and receptor-specific agonists was concentration-dependent when cells were treated for 30 min, prolonged stimulation of both receptors was essential to maintain a sustained ERK1/2 activation. More importantly, the agonist functions in modulating ERK1/2 phosphorylation in cotransfected cells were enhanced in serum-deficient conditions. Several reports have implicated a role for p38 MAPK pathway in different types of tumors [27-29]. The antiproliferative function of SSTR2 has been associated with increased p38 signaling, while in contrast, SSTR3 was devoid of such property [27]. In the heteromeric complex of human SSTR2/SSTR3, the activation of p38 in response to SSTR3-specific agonist uncovered a previously unnoticed role of SSTR3 on p38 MAPK. Taken together, the diverse functional response on MAPKs might be attributed to different duration of activities among various cell types, and SSTR-independent pathways [9,25,50].

We recently described the pronounced effect of oligomerization on the agonist mediated inhibition of cell proliferation in cotransfected cells expressing SSTR2/SSTR5 or SSTR4/SSTR5 when compared to monotransfectants [3,8]. Our results demonstrate significant antiproliferative effects upon activation of SSTR2/SSTR3 in cotransfected cells. Importantly, the activation of SSTR3 in cotransfectants displayed significant antiproliferative effect in comparison to monotransfected cells despite decreased cAMP inhibition. The antiproliferative functions of SSTR2/SSTR3 in cotransfectants were G_i_-dependent and might be exerted via modulation of ERK1/2 and p38 MAPK pathway which cannot be ruled out from the discussion.

In the current study, the activation of PARP-1 in the presence of SST and receptor-specific agonists in a pattern similar to p38 MAPK suggests a pro-apoptotic role for SSTR2/SSTR3. In agreement, TUNEL assay demonstrated a similar degree of cytotoxic response. Low expression of p21 and p27^Kip1^ has been often reported in tumor of various origins, and their up-regulation upon activation of SSTRs plays an important role in cell cycle arrest [3,4,22,30,51-55]. In line with this notion, an increased expression of p21 and p27^Kip1^ described here in response to SST and receptor-specific agonists suggests a cytostatic function for SSTR2/SSTR3. An earlier study attributed the sustained activation of ERK1/2 and p38 MAPKs to p21-mediated antiproliferation via SSTR2, whereas SSTR3 exhibited no effect on p21 and p38 signaling [27]. Also, SSTR2 mediated ERK1/2 activation has been linked to p27^Kip1^ mediated inhibition of cell proliferation [22]. In agreement with these observations, the sustained pERK1/2 and p38 signaling, together with induction of p21 and p27^Kip1^ might account for SSTR2/SSTR3 mediated antiproliferation in response to receptors activation.

In conclusion, our results show the modulation of intracellular signaling and antiproliferative functions by SSTR2 and SSTR3 attributed atleast in part to receptor heterodimerization. Our findings are of interest and might lead to identification of a novel therapeutic target in tumors expressing these receptor subtypes. To better understand the complexities of receptor functions, further studies are in progress in this direction.

## Abbreviations

cAMP: Cyclic adenosine monophosphate; PBS: Dulbecco’s phosphate buffered saline; ERK: Extracellular signal-regulated kinase; FSK: Forskolin; GPCRs: G protein-coupled receptors; HA: Hemagglutinin; HEK-293: Human embryonic kidney-293; MAPK: Mitogen-activated protein kinase; MTT: (3-(4, 5-Dimethylthiazol-2-yl)-2,5-diphenyltetrazolium bromide); PARP: Poly (ADP-ribose) polymerase; Pb-FRET: Photobleaching-fluorescence resonance energy transfer; SST: Somatostatin; SSTR: Somatostatin receptor; TUNEL: Terminal deoxynucleotidyl transferase dUTP nick end labeling.

## Competing interests

The authors declare that they have no competing interests.

## Authors’ contributions

UK designed the study. SAW performed the experiments and data analysis. UK and SAW wrote the manuscript. Both authors read and approved the final version of the manuscript.
